# The Effect of the Type and Colour of Placebo Stimuli on Placebo Effects Induced by Observational Learning

**DOI:** 10.1371/journal.pone.0158363

**Published:** 2016-06-30

**Authors:** Karolina Świder, Przemysław Bąbel

**Affiliations:** Jagiellonian University, Institute of Psychology, Kraków, Poland; University of Toledo, UNITED STATES

## Abstract

Research shows that placebo analgesia and nocebo hyperalgesia can be induced through observational learning. Our aim was to replicate and extend these results by studying the influence of the type and colour of stimuli used as placebos on the placebo effects induced by observational learning. Three experimental and two control groups were tested. All participants received pain stimuli of the same intensity preceded by colour lights (green and red) or geometric shapes (circles and squares). Before receiving pain stimuli, participants in the experimental groups, but not in the control groups, observed a model who rated pain stimuli that were preceded by either green lights (green placebo group), red lights (red placebo group), or circles (circle placebo group) as being less painful than those preceded by either red lights (green placebo group), green lights (red placebo group), or squares (circle placebo group). As a result participants in the experimental groups rated pain stimuli preceded by either green lights (green placebo group), red lights (red placebo group), or circles (circle placebo group) as being less painful than the participants in the control groups did, indicating that placebo effect was induced. No statistically significant differences were found in the magnitudes of the placebo effects between the three experimental groups (green placebo, red placebo, and circle placebo groups), indicating that neither the type nor the colour of placebo stimuli affected the placebo effects induced by observational learning. The placebo effects induced by observational learning were found to be unrelated to the individual differences in pain anxiety, fear of pain, and empathy.

## Introduction

Observational learning has been suggested as an explanatory mechanism for placebo effects [1], or as a process involved in the acquisition and modification of one of two main mechanisms that produce placebo effects, i.e., response expectancy [2]. The latter notion is in line with Bandura’s social learning theory, which postulates that observational learning results in the acquisition and modification of expectations [3].

Placebo effects that follow the administration of a placebo, can be divided into at least two different effects: placebo effect and nocebo effect, or–when placebo influences pain experience–placebo analgesia and nocebo hyperalgesia, respectively. We can talk about the placebo effect (and placebo analgesia), when an objectively neutral substance or procedure, e.g., a pill containing sugar, brings about positive results in the form of health improvement, i.e. reduction of the pain. On the other hand, we can talk about the nocebo effect (and nocebo hyperalgesia) when taking an objectively neutral substance or submitting oneself to a simulated medical procedure brings about negative effects in the form of health deterioration, e.g., greater sensitivity to pain.

Colloca and Benedetti [4] were the first to demonstrate experimentally that placebo analgesia can be induced by observational learning. They found that the magnitude of the placebo effect induced by observational learning was similar to the magnitude produced by verbal suggestion and classical conditioning combined, and it was significantly greater than the effect induced by verbal suggestion alone [4]. In a few subsequent studies it was also demonstrated that: (a) video-based observation of a model induced placebo analgesia similar in magnitude to observation of a live model [5]; (b) not only placebo analgesia, but also nocebo hyperalgesia could be induced by observational learning [6,7]; and (c) the sex of the model, but not the sex of the participant, influenced the magnitude of nocebo hyperalgesia, i.e., nocebo hyperalgesia was greater after a male than after a female model was observed [6].

In three out of four previous studies on the placebo effects induced by observational learning colour lights paradigm was applied [4–6]. Before receiving pain stimuli, the participants observed a model who rated pain stimuli preceded by green lights as less painful than those preceded by red lights. As a result of the observation participants rated pain stimuli preceded by green lights as significantly less painful than those preceded by red lights, although in fact they were receiving pain stimuli of the same intensity. In none of these previous studies the colours of the light stimuli associated with more and less pain were counterbalanced. It is then possible that the colour of the light stimuli may have biased the results of previous studies. It is suggestive that colours affect the perceived action of a drug and influence its effectiveness [8], e.g., red is associated with a stimulant effect, while green is associated with a tranquillizing effect. Moreover, the results of this previous research revealed that colour can act as a subtle environmental cue that has important influences on behaviour. This is especially true in the case of the colour red, which has been found to impair performance and evoke avoidance motivation [9]. Hence, the first and main aim of the study was to investigate the effects of the type and colour of stimuli used as placebos.

It has been shown that placebo analgesia induced by observational learning is positively correlated with empathy, but only in participants who observed a live model, and not in participants who watched a video recording of a model [5,10]. Similarly, it also has been demonstrated that the magnitude of the nocebo hyperalgesia is positively associated with empathy and negatively associated with personal distress in participants who observed a live model [6], but not in participants who watched a video recording of a model [7].

Vögtle and collaborators [7] also found that the nocebo hyperalgesia induced by observational learning is correlated with pain catastrophizing, but not with state and trait anxiety, or pain anxiety. These results seem to be contrary to the findings showing that participants with high levels of trait anxiety exhibit greater placebo and nocebo effects [11,12]. Thus, the second aim of the study was to extend these results by investigating the effects of empathy and both “pain anxiety” and “fear of pain” on the magnitude of the placebo effects induced by observational learning.

## Materials and Methods

### Participants

A total of 65 female volunteers participated in the study (mean age = 22.23±2.64) (Table 1). They were randomly assigned to five groups: three experimental groups (green placebo, red placebo, and circle placebo groups) and two control groups (control colours and control shapes groups). Each group consisted of 13 participants. All the participants were healthy and no one was taking any medication. They were informed that they were participating in a study on pain mechanisms and that they would receive 16 electrical pain stimuli. The participants gave their informed written consent to participate in the experiment. They were informed that they could stop participating at any point during the study without giving a reason. The study protocol was approved by the Research Ethics Committee at the Institute of Psychology of Jagiellonian University.

**Table 1:** Group means and standard deviations of all the study variables.

Group	*N*	Age	Pain NRS				IRI				PASS				FPQ-III		
			Stimuli type	Mean±*SD*	Stimuli type	Mean±*SD*	PT	PD	FS	EC	CA	EA	FA	PA	SP	Min. P	Med. P
Green placebo	13	21.81±2.63	Green	5.22±1.25	Red	6.00±0.87	15.9±3.9	14.7±4.7	17.5±3.4	19.5±3.9	26.0±7.1	25.6±5.1	14.6±8.0	15.0±8.4	34.4±5.1	20.2±6.7	26.5±5.9
Red placebo	13	21.71±1.61	Red	4.23±1.75	Green	4.84±1.74	19.4±4.3	14.8±5.0	16.0±2.7	18.8±4.2	18.8±9.5	19.4±9.5	10.8±5.6	14.4±8.8	32.4±7.8	17.5±4.0	23.±3.8
Circle placebo	13	22.90±4.30	Circle	4.46±1.35	Square	5.55±1.39	15.8±2.3	15.7±4.6	15.7±2.6	19.0±3.6	24.3±12.0	20.2±7.4	16.2±12.4	16.6±9.8	31.8±9.1	21.0±6.0	31.0±10.3
Control colours	13	22.75±1.95	Green	4.60±2.46	Red	4.65±2.49	16.4±2.4	15.3±5.0	15.6±1.8	18.8±3.7	19.8±7.8	20.9±6.0	9.6±5.0	10.4±6.7	36.8±5.9	19.8±6.2	27.8±6.5
Control shapes	13	21.75±1.83	Circle	4.38±1.42	Square	4.37±1.47	14.5±3.4	15.5±6.2	14.8±2.3	15.4±4.0	18.8±8.0	18.8±7.7	10.3±7.4	12.9±7.6	32.5±7.6	19.1±6.6	26.5±6.0

*N* = number of participants; Pain NRS = pain numeric rating scale; IRI = Interpersonal Reactivity Index; PT = Perspective Taking; PD = Personal Distress; FS = Fantasy Score; EC = Empathic Concern; PASS = Pain Anxiety Symptoms Scale; CA = Cognitive Anxiety; EA = Escape/Avoidance; FA = Fearful Appraisal; PA = Physiological Anxiety; FPQ-III = Fear of Pain Questionnaire-III; SP = Severe Pain; Min. P = Minor Pain; Med. P = Medical Pain.

### Stimuli

The pain stimuli were electric shocks delivered by the Constant Current High Voltage Stimulator (Digitimer, Welwyn Garden City, England, model DS7AH) to the inner side of the nondominant forearm through two durable stainless steel disk-electrodes that were eight mm in diameter and spaced 30 mm apart. Every participant received 16 pain stimuli of the same intensity (37 mA), with each stimulus lasting 200 μs. The intensity of the stimuli was determined according to the procedure previously used by Świder and Bąbel [6]. Successive electric shocks were separated by approximately 18-second intervals.

Two types of light and two types of geometric stimuli were used. In green placebo, red placebo, and control colours groups a total of eight red and eight green light stimuli were presented in full-screen mode on a computer screen (17 inches, resolution 1280 x 1024) facing the participant at a distance of approximately 50 cm. The colour light stimuli were full screen colour backgrounds. In circle placebo and control shapes groups, a total of eight white circles and eight white squares (both with white solid fill) were presented on black background in the middle of the computer screen (position on the X axis = 640 pixels; position on the Y axis = 512 pixels). Both geometric stimuli had similar dimensions (circle was inscribed within a square) and their size on the screen was 10 cm high and 10 cm wide. Each light or geometric stimulus was displayed for 15 seconds according to a predetermined pseudorandom sequence. Successive stimuli were separated by a 3-second black-light stimulus that was shown on the computer screen while a pain stimulus was applied. This prevented a pain stimulus from overlapping with the following light or geometric stimulus and allowed time for the participant to rate the pain intensity.

### Pain intensity scale and questionnaires

The participants rated pain intensity at the end of each stimulus on an 11-point numeric rating scale (NRS), ranging from 0 = no pain, to 10 = maximum imaginable pain. At the end of the experiment, the participants were asked to complete three questionnaires: the Interpersonal Reactivity Index (IRI) [13,14], the Pain Anxiety Symptoms Scale (PASS) [15], and the Fear of Pain Questionnaire-III (FPQ-III) [16].

The IRI is a 28-item measure of dispositional empathy that includes four subscales: Fantasy Score (FS; the tendency to transpose oneself imaginatively into the feelings and actions of fictitious characters in books, movies, and plays; e.g. “I daydream and fantasize, with some regularity, about things that might happen to me.”), Perspective Taking (PT; the tendency to adopt other people’s point of view; e.g. “I try to look at everybody's side of a disagreement before I make a decision.”), Personal Distress (PD; the tendency to experience the feelings of personal anxiety, discomfort, and unease in reaction to the others’ emotions, e.g. “In emergency situations, I feel apprehensive and ill-at-ease.”), and Empathic Concern (EC; the tendency to experience feelings of warmth, compassion, and concern for others; e.g. “I often have tender, concerned feelings for people less fortunate than me.”). Participants are asked to indicate their agreement with the items on a 5-point scale ranging from ‘Does not describe me well’ to ‘Describes me very well’. All four scales have satisfactory reliabilities–both internal (range from .71 to .77) and test-retest (from .62 to .71) [14].

As mentioned above, two different scales were used to measure pain anxiety and fear of pain. The PASS contains 40 items comprising four subscales: Cognitive Anxiety (CA; cognitive symptoms related to the experience of pain, such as racing thoughts or impaired concentration; e.g. “I feel disoriented and confused when I hurt.”), Escape/Avoidance (EA; overt behavioral responses to pain; e.g. “When I feel pain I try to stay as still as possible.”), Fearful Appraisal (FA; fearful thoughts related to the experience of pain or anticipated negative consequences of pain; e.g. “I think that pain is a signal that means I am damaging myself.”), and Physiological Anxiety (PA; physiological arousal related to the experience of pain; e.g. “I become sweaty when in pain.”). All items are rated on a frequency scale from 0 (never) to 5 (always). The alpha coefficients ranged from .81 to .94 [15].

The FPQ-III is a 30-item measure that consists of three subscales: Severe Pain (SP; e.g. “Being in an automobile accident.”), Minor Pain (Min. P; e.g. “Biting your tongue while eating.”), and Medical Pain (Med. P; eg. “Having a blood sample drawn with a hypodermic needle.”). Items are scored on a 5-point scale ranging from 1 (not at all) to 5 (extreme). Internal consistency and test-retest reliability of the FPQ-III were found to be good and ranged from .88 to .92 and from .69 to .76, respectively [16].

### Design and procedures

#### Experimental groups

The study consisted of three phases for the experimental groups (i.e. green placebo, red placebo, and control colours groups): (1) an observation phase, (2) a pain stimuli phase, and (3) a testing phase. During the first phase of the study, 3 people were present in the laboratory: a female experimenter delivering the pain stimuli, a female model (one of two), and a female participant. Both models were 22 year-old psychology students in their last year at the Institute of Psychology at Jagiellonian University. Before the study commenced, the models were trained to simulate the experimental procedure, i.e., to rate pain appropriately because no pain stimuli were administered to them. Although our previous study showed that nocebo hyperalgesia was greater after a male than after a female model was observed [6], the models in this study were females only. In this way we wanted to exclude any interaction effects of the sex of the model and the sex of the participants on the results of the study.

**Fig 1:**
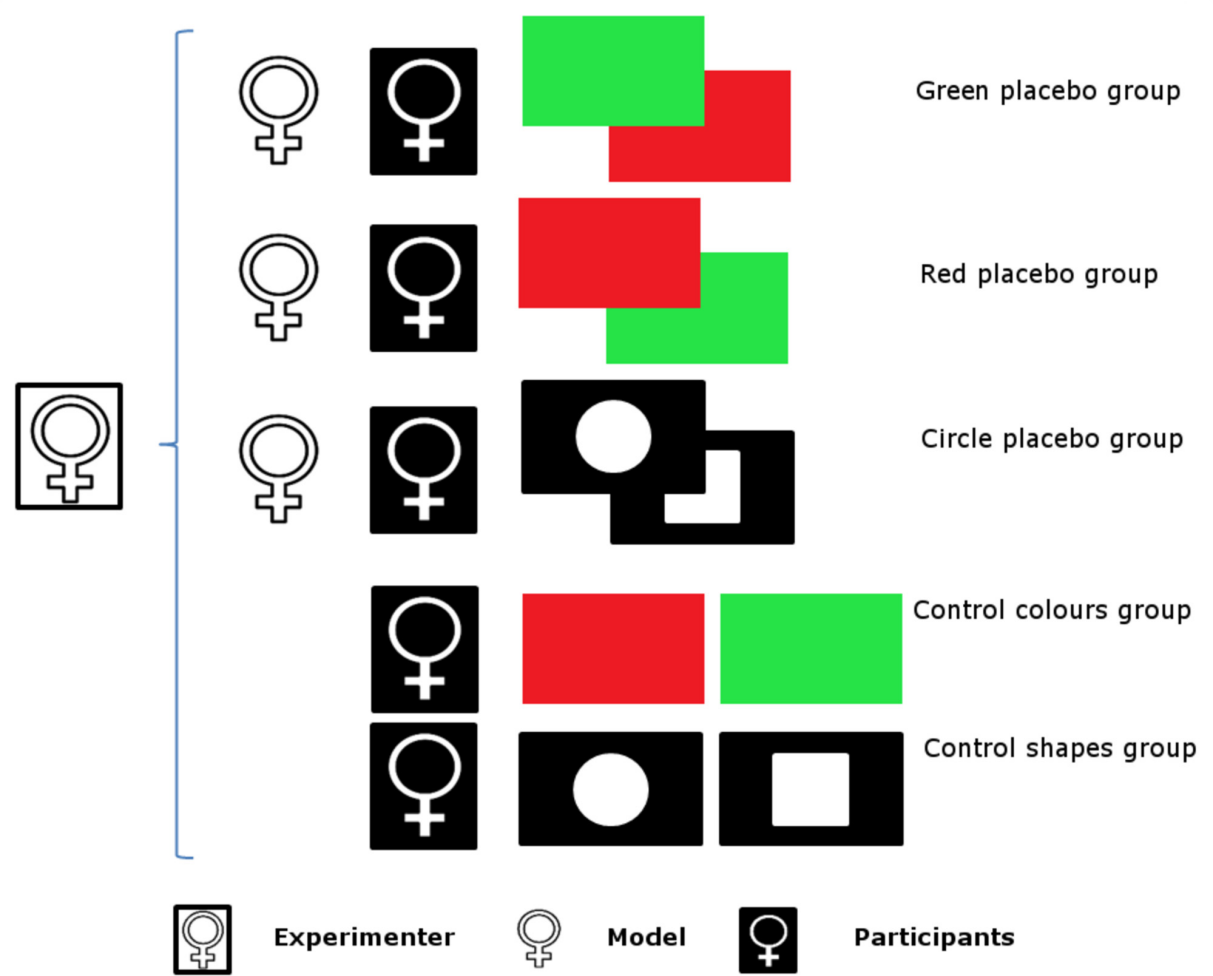
Study design. Participants in experimental groups observed a model who rated pain stimuli preceded by green lights (green placebo group), red lights (red placebo group) or circles (circle placebo group), as being less painful than those preceded by red lights (green placebo group), green lights (red placebo group), or squares (circle placebo group). All participants received 16 pain stimuli preceded by eight green lights and eight red lights (green placebo and red placebo groups) or eight circles and eight squares (circle placebo group). Participants in the control colours and control shapes groups did not observe a model before they received the 16 pain stimuli preceded by eight green lights and eight red lights (control colours group) or eight circles and eight squares (control shapes group).

When the participant arrived at the laboratory, she was informed that she would need to wait for a short time because the next participant scheduled to take part in the study had asked to be tested first because she was pressed for time. The next participant was in fact a model, who then simulated receiving 16 pain stimuli preceded by eight green and eight red lights (in green placebo and red placebo groups) or eight white circles and eight white squares (in circle placebo group), according to a pseudorandom sequence. Electrodes were applied to the inner side of the nondominant forearm of the model, and she rated aloud the intensity of each of the apparent pain stimuli according to the following scheme. In green placebo group, the stimuli that were preceded by green lights were rated as 2 to 4 on the NRS, whereas those that were preceded by red lights were rated as 7 to 9. Thus in green placebo group the model simulated an analgesic effect for the green light, which acted as a placebo. The opposite scheme was applied in red placebo group. The stimuli that were preceded by red lights were rated as 2 to 4 on the NRS, whereas those that were preceded by green lights were rated as 7 to 9. Thus in red placebo group the model simulated an analgesic effect for the red light, which acted as a placebo. In circle placebo group, the stimuli preceded by circles were rated as 2 to 4 on the NRS, whereas those that were preceded by squares were rated as 7 to 9. Thus in circle placebo group the model simulated an analgesic effect for the circle, which acted as a placebo (Fig 1).

The participant was sitting behind the model and observing her simulation of the experimental session. To ensure that the participant’s attention was kept constant throughout the first phase of the experiment, she was asked to help the experimenter by noting the colour (in green placebo and red placebo groups) or shape (in circle placebo group) of each of the stimuli presented to the model and the value of each of the pain ratings reported by the model. The participants therefore had an opportunity to associate one type of stimulus with less intense pain (green lights in green placebo group, red lights in red placebo group, and circles in circle placebo group) and the other type of the stimulus with more pain (red lights in green placebo group, green lights in red placebo group, and squares in circle placebo group). This observational phase lasted approximately five minutes.

After the first phase of the experiment, the participants underwent the same experimental session (phase 2: the pain stimuli phase). They received 16 pain stimuli preceded by eight green-light and eight red-light stimuli (in green placebo and red placebo groups) or eight circles and eight squares (in circle placebo group) in the same pseudorandom sequence as the models; this sequence was the same for all the participants. However, the participants received real electrical pain stimuli, each of the same intensity (37 mA, 200 μs duration). After receiving each of the stimuli, the participants noted the colour of the light stimulus (in green placebo and red placebo groups) or the shape of the stimulus (in circle placebo group) that preceded the pain stimulus and rated the intensity of the pain stimulus using the NRS. When the experimental session (i.e., the pain stimuli phase) was finished, the participants were asked to complete the IRI, the PASS, and the FPQ-III; this was the testing phase, which lasted approximately 15 minutes.

#### Control groups

Participants in the control groups (i.e., control colours and control shapes groups) underwent only phases 2 and 3 of the study: the pain stimuli phase and the testing phase. They did not observe a model before the experimental session started. Participants in control colours group received 16 pain stimuli preceded by 8 green-light and 8 red-light stimuli in the same pseudorandom sequence that was used for both models and all the participants in the experimental groups. The procedure was the same in control shapes group as it was in control colours group, with one exception: 16 pain stimuli were preceded by eight circles and eight squares. This methodological approach allowed us to assess the effects of the colour of the light stimuli, as well as the effects of the shape of the geometric stimuli, on the pain ratings. The procedure in phase 2 was exactly the same for the participants in control colours and control shapes groups as it was for the participants in green placebo, red placebo, and circle placebo groups: the experimental session lasted approximately five minutes.

#### Statistical analysis

To address the first aim of the study, i.e. to investigate the effects of the type and colour of stimuli used as placebos, one-way analysis of variance (ANOVA) was performed for the difference between the placebo and nonplacebo pain ratings, with group (green placebo, red placebo, circle placebo, control colours, and control shapes) as a between-subject factor. *F*-tests were followed by planned comparison tests. To test whether observational learning had an effect on the difference between the placebo and nonplacebo pain ratings, the three experimental groups (model condition) were compared with the control groups (no-model condition). Specifically, green placebo, red placebo, and circle placebo groups were compared with control colours and control shapes groups. To determine whether the type of the stimuli used as a placebo had an effect on the difference between the placebo and nonplacebo pain ratings induced by observational learning, the three experimental groups (model condition) were compared with one another. Specifically, green placebo group was compared with red placebo and circle placebo groups, and red placebo group was compared with circle placebo group. To test whether there is a difference between the control groups as a function of stimuli type, control colours group was compared with control shapes group.

To address the second aim of the study, i.e. to investigate the effects of empathy and both “pain anxiety” and “fear of pain” on the magnitude of the placebo effects induced by observational learning, Pearson product-moment correlation coefficients (*r*) were calculated between the difference between the placebo and the nonplacebo pain ratings separately in the experimental (model) condition (green placebo, red placebo, and circle placebo groups) and in the control groups (control colours and control shapes groups) and each of the other variables: FS, PT, PD, EC, CA, EA, FA, PA, SP, Min. P, and Med. P. Pearson product-moment correlation coefficients (*r*) were also calculated to examine the relationships between the models’ and participants’ pain ratings.

All the analyses were conducted using the STATISTICA data analysis software system version 10 (StatSoft Inc., Tulsa, OK, USA). The level of significance was set at *p* < 0.05, but the *p*-values were adjusted by Bonferroni correction whenever needed.

## Results

**Fig 2:**
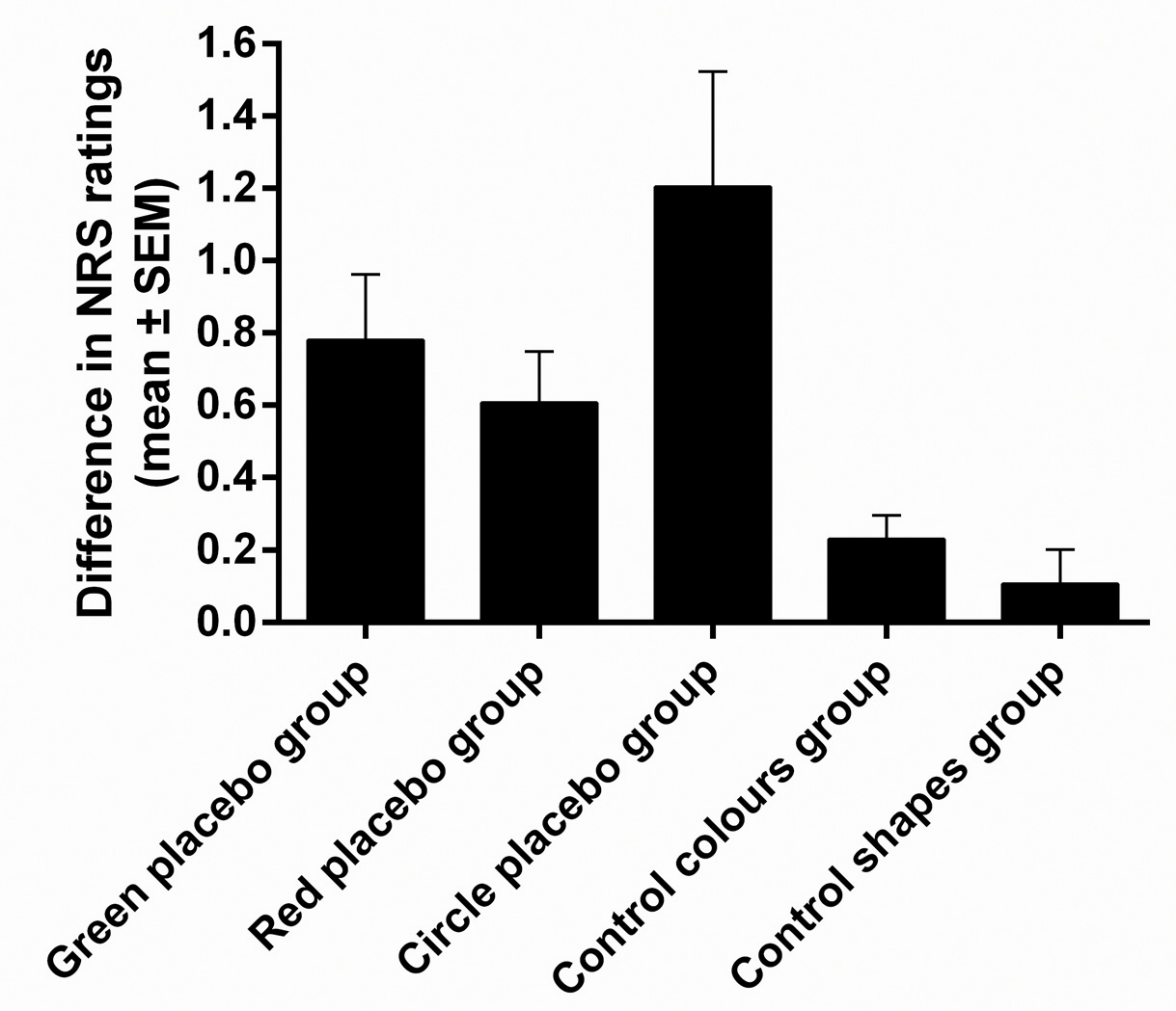
Differences between placebo and nonplacebo pain ratings in the three experimental groups (model condition; green placebo, red placebo, and circle placebo groups) and two control groups (no-model condition; control colours and control shapes groups). The difference between placebo and nonplacebo pain ratings in the model condition was significantly higher than in the no-model condition. However, there were no statistically significant differences between the three experimental groups as well as between the two control groups.

**Fig 3:**
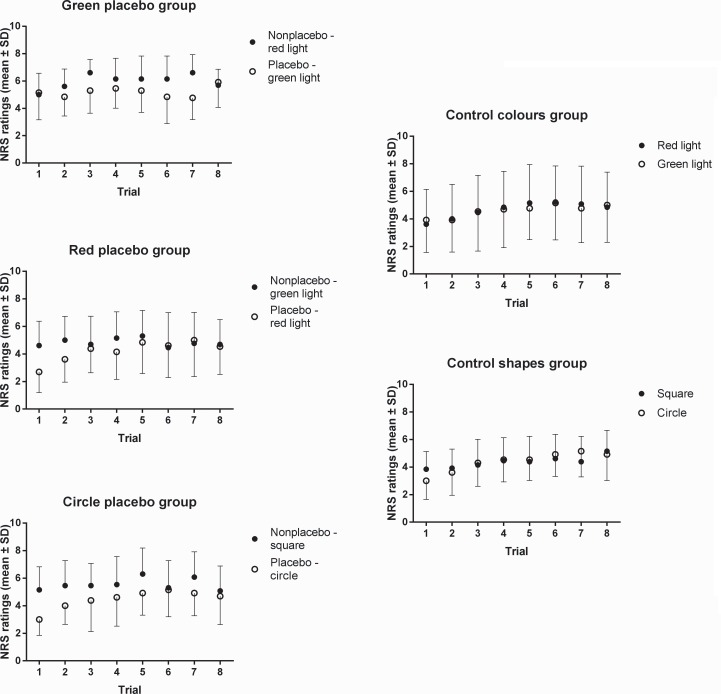
Trial by trial placebo versus nonplacebo mean pain ratings of the three experimental groups (model condition; green placebo, red placebo, and circle placebo groups) and two control groups (no-model condition; control colours and control shapes groups). In the model condition, as opposed to the no-model condition, pain stimuli preceded by placebo stimuli (i.e., green lights in green placebo group, red lights in red placebo group, and circles in circle placebo group) were rated as less painful than pain stimuli that were preceded by nonplacebo stimuli (i.e., red lights in green placebo group, green lights in red placebo group, and squares in circle placebo group).

ANOVA revealed a statistically significant main effect of group (*F*_(4,60)_ = 5.71, *p* < .001, *ŋ*^*2*^ = .28). A planned comparison test revealed that the difference in placebo and nonplacebo pain ratings in the model condition (green placebo, red placebo, and circle placebo groups) was significantly greater than in the no-model condition (control colours and control shapes groups; *F*_(1,60)_ = 19.73, *p* < .001, *ŋ*^*2*^ = .24). Planned comparison tests showed that there was no statistically significant differences between green placebo and red placebo groups (*F*_(1,60)_ = .39, *p* > .05, *ŋ*^*2*^ = .003), green placebo and circle placebo groups (*F*_(1,60)_ = 1.22, *p* > .05, *ŋ*^*2*^ = .02), and red placebo and circle placebo groups (*F*_(1,60)_ = 2.97, *p* > .05, *ŋ*^*2*^ = .05). Moreover, there was no statistically significant difference between control colours and control shapes groups (*F*_(1,60)_ = .06, *p* > .05, *ŋ*^*2*^ = .001) (see Figs 2 and 3).

No statistically significant correlation was found between participants’ and models’ NRS pain ratings (*r* = -.06, *p* > .05), indicating that the participants’ pain ratings were independent from models’ pain ratings. Only the results of one of the FPQ-III subscales, i.e. Medical Pain, was found to correlate modestly (*r* = .36) with the difference between the placebo and the nonplacebo pain ratings in the control groups (control colours and control shapes groups) rather than in experimental (model) condition (green placebo, red placebo, and circle placebo groups) (see Table 2). However, after the Bonferroni correction was applied, this correlation was not statistically significant.

**Table 2:** Pearson product-moment correlation coefficients (*r*) between questionnaires scores and the difference between the placebo and the nonplacebo pain ratings in the experimental (model) condition (green placebo, red placebo, and circle placebo groups) and in the control groups (control colours and control shapes groups).

The difference between the placebo and the nonplacebo pain ratings	IRI				FPQ-III			PASS			
	PT	PD	FS	EC	SP	Min. P	Med. P	CA	EA	FA	PA
Experimental groups	-.09	.03	-.06	.1	-.13	-.04	-.012	-.21	-.25	-.15	-.16
Control groups	-.09	.14	.21	.18	.28	.15	.36	.03	-.11.	.16	.01

NRS = numeric rating scale; IRI = Interpersonal Reactivity Index; PT = Perspective Taking; PD = Personal Distress; FS = Fantasy Score; EC = Empathic Concern; PASS = Pain Anxiety Symptoms Scale; CA = Cognitive Anxiety; EA = Escape/Avoidance; FA = Fearful Appraisal; PA = Physiological Anxiety; FPQ-III = Fear of Pain Questionnaire-III; SP = Severe Pain; Min. P = Minor Pain; Med. P = Medical Pain.

## Discussion

Our study showed that there was a significantly greater difference between placebo- and nonplacebo pain ratings in participants who had observed a model rating pain stimuli preceded by placebo stimuli (i.e. green lights in green placebo group, red lights in red placebo group, and circles in circle placebo group) as significantly less painful than pain stimuli preceded by nonplacebo stimuli (i.e. red lights in green placebo group, green lights in red placebo group, and squares in circle placebo group), even though they were receiving pain stimuli of the same intensity. In this way we replicated the results of the previous studies demonstrating that placebo effects can be induced by observational learning [4–7]. Moreover, our study showed that placebo effects can be induced by observational learning regardless of the type and colour of stimuli used as placebos. Specifically, we did not find any differences in the magnitudes of the placebo effects induced by observational learning when either red lights, green lights, or circles served as placebos. As the widespread acceptance of the colour lights paradigm proposed by Colloca and Benedetti [17] increases (it has been applied in several studies on the mechanisms of placebo effects [4–6,10,18]), the results of our study may also be relevant to that paradigm.

Similarly to our previous study [6], the colour of the light (green in green placebo group; red in red placebo group) or the shape (circle in circle placebo group) was the only placebo in our study. We did not use any other intervention, such as a special electrode applied to the middle finger that was used in the two previous studies [4,5]. However, even if no inert treatment is administered, the effect of suggestions and expectations of improvement or exacerbation can be called a placebo- or nocebo-related effect [19]. In our study modelling (i.e. rating pain stimuli preceded by one colour or shape as more painful than those preceded by the other colour or shape) indeed was a kind of suggestion and a mean by which expectations concerning painfulness of the stimuli may have been acquired [2]. We used visual stimuli without any other intervention as placebos because we did not want to confound those two different factors that might have been affected by modelling. However, as this procedure was found to be as effective as the one used in the two previous studies [4,5] it is suggested that the results of our study are generalizable to the effects of modelling on both visual stimuli and inert treatment.

We did not find any significant correlations between either pain anxiety or fear of pain difference between the placebo and the nonplacebo pain ratings in experimental groups, as well as in control groups. This is in line with the results of one of the previous studies which found that nocebo hyperalgesia induced by observational learning is not correlated with state and trait anxiety, or pain anxiety [7].

Previous studies have found that individual differences in empathy are related to the placebo effects induced by observational learning, but only when the participants observed a live model [5,6,10]. When participants watched a video recording of a model, their empathy was found to be unrelated to the placebo effects induced by observational learning [4,7]. The current study did not find any relationship between empathy and pain ratings even though the participants observed a live model. This difference may be due to the more subtle procedure of observational learning used in the current study than in two other studies in which strong correlations were found between empathic concern and placebo analgesia [4,6]. In other words, we led participants to believe that their observation of the model was a matter of chance rather than a designed part of the study. This explanation is in line with the results of a previous study in which the same subtle procedure was used and a very weak relationship was found between empathy and the magnitude of nocebo hyperalgesia [6].

Some limitations of our study should be acknowledged. First, acute experimental pain was studied and the results may not be generalizable to clinical pain, especially chronic pain. Second, similarly to previous studies on placebo effects induced by observational learning [4–7], only females participated in the study, and in view of gender differences in pain perception [20,21], the results may not be generalizable to males. On the other hand, as only females participated in all of the previous studies on placebo effects induced by observational learning, the results of our study can be related to the results of the previous research as they are not biased by gender differences in pain perception. Third, the models were all female and previous research showed that nocebo hyperalgesia was greater after a male rather than a female model was observed [6]. Fourth, we did not control for state anxiety, which may have mediated the results of the current study, given that state anxiety has been found to influence both analgesic and hyperalgesic placebo effects [12,22–26]. Fifth, sample size was rather small (13 persons in each group), however not much smaller than in the previous studies on the placebo effects induced by observational learning in which similar methodology (colour lights paradigm) was applied [4–6]. Sixth, similarly to previous studies using a colour light paradigm to study placebo effects, like that proposed by Colloca and Benedetti [17], shapes were not counterbalanced. On the other hand, colours were counterbalanced between green placebo and red placebo groups. However, it does not seem to be crucial in light of our results.

Our study appears to be one of a very few to investigate observational learning as a mechanism for producing placebo effects, and the first to attempt to determine the effect of the type and colour of stimuli used as placebos on the placebo effects induced by observational learning. We replicated the results of the previous studies demonstrating that placebo effects can be induced by observational learning [4–7]. We also extended previous findings by showing that neither the type nor the colour of stimuli used as placebos affects the placebo effects induced by observational learning. Although we failed to replicate the findings that empathy is related to the placebo effects induced by observational learning, our study supports and extends the previous findings by demonstrating that individual differences in pain anxiety and fear of pain are not related to the effects of observational learning on pain ratings.

Although the results of our study do not support the conclusion that the type and colour of placebo stimuli affect the placebo effects induced by observational learning, one cannot exclude that future research will find evidence for such an effect. Moreover, although our study showed that green light, red light, and circle are similarly effective in inducing placebo effects by observational learning, future research should address the question whether there are any stimuli or classes of stimuli that would be particularly effective in producing placebo effects in patients who receive a variety of coloured and differently shaped pills.
